# Endophytic Actinomycetes: A Novel Source of Potential Acyl Homoserine Lactone Degrading Enzymes

**DOI:** 10.1155/2013/782847

**Published:** 2013-02-04

**Authors:** Surang Chankhamhaengdecha, Suphatra Hongvijit, Akkaraphol Srichaisupakit, Pattra Charnchai, Watanalai Panbangred

**Affiliations:** ^1^Department of Biology, Faculty of Science, Mahidol University, Rama VI Road, Bangkok 10400, Thailand; ^2^Mahidol University-Osaka University Collaborative Research Center for Bioscience and Biotechnology (MU-OU:CRC), Bangkok, Thailand; ^3^Faculty of Science, Mahidol University, Rama VI Road, Bangkok 10400, Thailand; ^4^Center of Excellence on Agricultural Biotechnology (AG-BIO/PERDO-CHE), Bangkok, Thailand; ^5^Department of Biotechnology, Faculty of Science, Mahidol University, Rama VI Road, Bangkok 10400, Thailand

## Abstract

Several Gram-negative pathogenic bacteria employ *N*-acyl-L-homoserine lactone (HSL) quorum sensing (QS) system to control their virulence traits. Degradation of acyl-HSL signal molecules by quorum quenching enzyme (QQE) results in a loss of pathogenicity in QS-dependent organisms. The QQE activity of actinomycetes in rhizospheric soil and inside plant tissue was explored in order to obtain novel strains with high HSL-degrading activity. Among 344 rhizospheric and 132 endophytic isolates, 127 (36.9%) and 68 (51.5%) of them, respectively, possessed the QQE activity. The highest HSL-degrading activity was at 151.30 ± 3.1 nmole/h/mL from an endophytic actinomycetes isolate, LPC029. The isolate was identified as *Streptomyces* based on *16S* *rRNA* gene sequence similarity. The QQE from LPC029 revealed HSL-acylase activity that was able to cleave an amide bond of acyl-side chain in HSL substrate as determined by HPLC. LPC029 HSL-acylase showed broad substrate specificity from C_6_- to C_12_-HSL in which C_10_HSL is the most favorable substrate for this enzyme. In an *in vitro* pathogenicity assay, the partially purified HSL-acylase efficiently suppressed soft rot of potato caused by *Pectobacterium carotovorum* ssp. *carotovorum* as demonstrated. To our knowledge, this is the first report of HSL-acylase activity derived from an endophytic *Streptomyces*.

## 1. Introduction

Bacterial pathogens infecting plants, animals, and humans cause a tremendous economic loss worldwide. Conventional treatments, such as application of antibacterial agents, significantly contribute to selection of resistant microorganisms as well as environmental contamination [1]. A number of concerns on using antibiotics have brought investigators to search for alternative strategies to combat with these pathogens. Attenuation of virulence phenotypes rather than killing of causative agents has gained an interest since this strategy does not introduce selective pressure which potentially leads to development of antibiotic-resistant bacteria [2]. Certain bacterial pathogens regulate their virulence by monitoring population density which is known as quorum sensing (QS) mechanism [3–5]. In Gram-negative bacteria, QS is often mediated by *N*-acyl-L-homoserine lactone (HSL) signal molecules [4]. Interference of HSL-dependent QS, commonly known as quorum quenching (QQ), has been demonstrated to be an effective antimicrobial strategy for controlling virulent pathogens [6–9]. QQ can be achieved by degradation of QS signal molecules by enzymatic digestion, significantly decreasing functions of signaling molecules. The QQ enzymes (QQEs) have been reported from several Gram-positive bacteria including *Arthrobacter* sp. IBN110, *Bacillus *sp. 240B1, *Geobacillus kaustophilus* strain HTA426, *Mycobacterium avium* subsp. *paratuberculosis* K-10, *Solibacillus silvestris* StLB046 [10], and* Streptomyces* M664 [8]. However, there are differences in the catalytic spectrum among QQEs toward HSLs. In the search for QQE-producing bacteria, actinomycetes are of great interest since they possess an ability to produce and secrete various extracellular hydrolytic enzymes [11, 12]. A number of actinomycetes have been isolated from several natural sources, including rhizospheric soil and plant tissues. Biological functions of actinomycetes predominantly depend on sources from which the bacteria are isolated. Natural resources in megabiodiversity with high selective pressure and microbial competition in tropical regions are well recognized as an important resource of new anti-microbial agents as well as QQE [13].

To date, the isolation of actinomycetes with high QQE activity from endophytes has never been reported. Screening for QQE-producing actinomycetes in this study showed the diversity and abundance of HSL-degrading actinomycetes from soil and plant tissues for the first time. The QQE from the high activity strain was shown to hydrolyze HSL with a broad range of chain length from *N*-hexanoyl-L-homoserine lactone (C_6_HSL) to *N*-dodecanoyl-L-homoserine lactone (C_12_HSL); hence, this enzyme could be potentially used to attenuate virulence of a broad range of bacterial pathogens with different QS signal molecules. 

## 2. Materials and Methods

### 2.1. Isolation of Rhizospheric and Endophytic Actinomycetes

 Rhizospheric soil samples were collected from various provinces in Thailand, including Bangkok, Chanthaburi, Nongbualumpoo, Prachinburi, and Rayong provinces. To isolate rhizospheric actinomycetes, 1.0 g of soil was serially diluted in 4 mL of 0.85% normal saline solution (NSS). The appropriate dilution was spread on selective agars, which are Pridham's and water proline supplemented with 25 *μ*g/mL nalidixic acid and 50 *μ*g/mL cyclohexamide [14]. Isolates with typical actinomycetes colony morphology were selected for screening of HSL-degrading activity. In order to isolate endophytic actinomycetes, leaves, fruits, seeds, and stems of plant samples were collected from various regions in Thailand including Bangkok, Salaya botanical garden in Nakhon pathom, and Nam Nao national park in Phetchabun. All dirt was removed by running tap water. Samples were air-dried at room temperature for one week. Plant samples were cut into small pieces. Surface sterilization was performed by treating with 10% sodium hypochlorite (MERCK), 70% ethanol and followed by washing twice with sterile water. The samples were then air-dried and placed on water agar medium (containing only 1.5% agar) supplemented with 25 *μ*g/mL nalidixic acid and 50 *μ*g/mL cyclohexamide. Actinomycetes colonies were selected for further study.

### 2.2. HSL Inhibition Assay

Actinomycetes isolates were grown on Waskman's agar (1% glucose, 0.5% peptone, 0.5% meat extract, 0.3% NaCl, and 1.2% agar) for 3 days, subcultured in 301 broth (2.4% starch, 0.1% glucose, 0.3% peptone, 0.5% meat extract, and 0.3% CaCO_3_), and further incubated at 28°C with shaking for 6 days. Forty microliters of cell-free supernatant was mixed with an equal volume of 40 *µ*M* N-*decanoyl-L-homoserine lactone (C_10_HSL) in 0.1 M Tris-HCl pH 6.8. The mixture was incubated at 28°C for 1 h with gentle agitation and the reaction was stopped by heating at 95°C for 5 min. Then 10 *µ*L of reaction mixture was used to determine the remaining of C_10_HSL on a bioassay plate. For determination of HSL-degrading activity, *Agrobacterium tumefaciens* NTL4 (pZLR4) (kindly provided by Professor Stephen K. Farrand, Department of Crop Sciences and Microbiology, University of Illinois, USA) was used as a biosensor strain in bioassay to long-chain HSL (C_8_ to C_12_HSL) [15].* Chromobacterium violaceum* CV026 (kindly provided by Professor Paul Williams, Department of Molecular Microbiology, University of Nottingham, UK) was used to detect *N*-butanoyl-L-homoserine lactone (C_4_HSL), *N*-hexanoyl-L-homoserine lactone (C_6_HSL), and 3-oxohexanoyl-L-homoserine lactone (3-oxo-C_6_HSL) [16]. Each reporter strain was cultivated overnight in nutrient broth (NB) and Luria-Bertani broth (LB), respectively. To determine HSL-degrading activity qualitatively, an overnight culture of the biosensor strain (2.5 mL) was mixed with 5 mL of agar. The mixture was overlaid on an AB minimal medium bioassay plate [17] supplemented with 40 *µ*g/mL of 5-bromo-4-chloro-3-indolyl-D-galactopyranoside (X-gal) for *A. tumefaciens* NTL4 (pZLR4) and Luria-Bertani agar (LA) for *C. violaceum* (CV026). A well on an agar plate was made by punching with a cork borer (*∅* = 0.4 cm). Ten microliters of the heat inactivated reaction mixture was dropped in each well. All plates were incubated at 30°C overnight to allow color zone developing. Blue and purple color zones were developed around colonies of *A. tumefaciens* NTL4 (pZLR4) and* C. violaceum* (CV026), respectively, by the induction of the residual HSL in the reaction mixture. The residual amounts of HSL were calculated using relationship equations based on the color zone size and known amounts of HSL [6]. Relative activity of HSL-degrading enzyme was calculated by using the following formula: relative HSL-degrading activity = (HSL-degrading activity/initial amount of substrate) × 100. For quantitative HSL-degrading activity determination, bioassay agar medium in the plate was cut into separated slices across the plate (1 cm in width). Five microliters of the reaction mixture was added to one end of an agar slice and then the culture of biosensor strain at an OD_600_ of 1 was progressively spotted (0.6 *µ*L per spot) at further distances from the loaded reaction mixture. The last induced color colony to the origin of the reaction mixture sample in each agar slice was measured. Amounts of HSL in the reaction mixture were determined based on relationship equation of adding known amounts of HSL to the bioassay slices and determining the distance of the color colony from the origin. HSL-degrading activity reported as nmole/h was calculated from subtraction of the initial amount of HSL substrate with leftover amount from the enzymatic digestion. 

### 2.3. Strain Identification

The *16S rRNA* gene of isolate LPC029 was amplified using conserved primers [18]. PCR was performed for 30 cycles at 95°C for 45 seconds, 45°C for 45 seconds, and 72°C from 1.5 minutes. The PCR product was sequenced by the dideoxy chain-termination method [19].

### 2.4. Preparation of Partially Purified HSL-Degrading Enzyme

The endophytic isolate LPC029 was harvested after 6 days of culture in 1,000 mL 301 medium by centrifugation at 4°C (10,000 g) for 10 min. The supernatant was filtered through a 0.45 *μ*m membrane and stored at 4°C. Ammonium sulfate was added to the culture supernatant to achieve 60% saturation and the solution was left at 4°C for 16 h. The precipitate was collected by centrifugation, dissolved, in 6 mL of 20 mM sodium phosphate buffer pH 7.0, and dialyzed (dialysis tubing, MW cut-off 10 kDa; Sigma, USA) overnight against the same buffer. The dialyzed sample was then collected and stored at 4°C.

### 2.5. HSL Degradation Assay

 C_10_HSL was used as a substrate in the determination of hydrolytic products of the HSL-degrading enzyme. Substrates were prepared as a stock solution at 8 mM in 80% ethanol. The HSL-degrading product was analyzed by mixing 13 *µ*g of the partially purified HSL-degrading enzyme and 350 mM of C_10_HSL in 700 mL of 20 mM sodium phosphate buffer pH 7.0. After incubation at 30°C for 16 h, the reaction mixture was extracted three times with equal volumes of ethyl acetate. The organic phase was evaporated to dryness at 40°C by a rotary evaporator. The dried sample was dissolved in 50 *µ*L of HPLC grade methanol. Five microliters of HSL-degraded products was introduced onto Shiseido Capcell Pak C18 (4.6 × 250 mm I.D.; particle size 5 *µ*m) previously equilibrated with 10% acetonitrile. The elution was performed with linear gradient of 10–90% acetonitrile, at a flow rate of 1 mL/min under the following condition: 10% acetonitrile from 0 to 8 min; linear gradient to 90% acetonitrile from 8 to 35 min. Homoserine lactone (3.5 mM) and 35 mM of C_10_HSL were used as a standard. HPLC chromatogram of the products was analyzed by diode array detector with UV ranging from 200 to 280 nm (Agilent 1200 HPLC). To check substrate specificity, HSL-acylase was reacted with different HSL-substrates including C_4_HSL, C_6_HSL, 3-oxo-C_6_HSL, 3-oxo-C_8_HSL, C_10_HSL, and C_12_HSL. Reaction mixture was composed of 0.4 mM each of HSL substances in 20 mM sodium phosphate at pH 7.0 and 2 *µ*g of the partially purified HSL-degrading enzyme in 50 *µ*L. The mixture was incubated at 30°C for 1 h. The HSL-substrate that remained in the reaction mixture was determined by the bioassay method as described above.

### 2.6. *In Vitro* Pathogenicity Assay

The assay was performed as described by Burr et al. [20]. The potatoes' tubers of about the same size were washed with tab water and pretreated in 5% sodium hypochlorite (Merck) for 10 min then soaked with sterile water. The tubers were dried in a laminar flow cabinet.* P. carotovorum* ssp. *carotovorum* (*Pcc*) was cultured in 50 ml of LB broth at 30°C overnight. The culture at OD_600_ of 1 was further serially diluted with 0.85% NSS to 10^−5^. Ten microliters of 100,000-folded dilution of *Pcc* (ca. 1000 cfu/10 *µ*L) was mixed with 2 *µ*g of partially purified HSL-degrading enzyme. The reaction was then further incubated at 30°C for 1 h and was inoculated into a potato tuber. A two-hundred-microliter pipette tip was used to punch a hole of 22 mm in depth, 2 holes per tuber. The two holes in each potato tuber were each filled with 0.85% NSS (negative control), 1000 cells of *Pcc* (positive control), the mixture of *Pcc* and HSL-acylase, and HSL-acylase alone. The inoculated potato tubers were further sealed with a sterilized sticker. To make a moisture condition, the tubers were wrapped with aluminum foil and sterile moist towels. The wrapped-potato tubers were incubated in a closed box at 30°C for 3 days.

## 3. Results and Discussion

### 3.1. Screening of HSL-Degrading Actinomycetes and HSL-Degrading Activity

The abundance and diversity of HSL-degrading actinomycetes isolated from soil and plant tissues were assessed. They were isolated on the basis of their typical morphologies (filamentous growth, spore chain, and several types of convex and margin colonies on selective medium) according to Bergey's Manual of Systematic Bacteriology [21]. While 344 actinomycetes could be isolated from 43 rhizospheric soil samples, only 132 isolates were obtained from 64 plant samples. Among these actinomycetes isolates, the number of isolates with HSL degrading activity was found in higher frequency in endophytic isolates (51.5%) than in rhizospheric isolates (36.9%) (Table 1). While several previous studies have indicated the HSL-degrading activity in soil bacteria [8, 22, 23], this study is the first report of such activity in endophytic actinomycetes. The evolution race for survival in tropical ecosystem reinforces strong competition among organisms, which may result in a plethora of chemical molecules [24], and enzymes [11, 12] with biological functions. Consequently, there is a high probability that microorganisms associated with tropical plants might be a source of bioactive compounds and enzymes. Among our isolates with HSL-degrading activity, 4 rhizospheric actinomycetes (PS032, SWP036, SWP042, and SWP043) and 5 endophytic actinomycetes (LPC026, LPC029, PC005, PC052, and PC053) were found to be the potential strains in HSL degrading as they were able to degrade HSL of greater than 96.9% relative activity (Table 1). Quantitative measurement of enzymatic activity among these 9 isolates showed that isolate LPC029 from *Gmelina arborea *Roxb. possessed the highest HSL-degrading activity at 151.30 ± 3.1 nmole/h/mL (Table 2). Strain LPC029 was later identified with *16S rRNA* gene sequence (GenBank accession number KC153060) to be closely related to *S. globisporus* at 99% homology. The presence of HSL-degrading activity in both soil and plant isolates may be due to their common need of HSL-degraded product as carbon and energy sources [25] and for competition with other microbes to protect their ecological niche [26]. In addition, endophytic bacteria have previously been reported to be involved in several biological functions such as promoting plant growth, biocontrol agents, and phytoremediation [24, 27–29]. These functions stem from the production of their natural compounds and antipathogen metabolites inside the host plant [29].

### 3.2. HPLC Analysis of C_10_HSL Hydrolysis by LPC029 HSL-Degrading Enzyme

**Figure 1:**
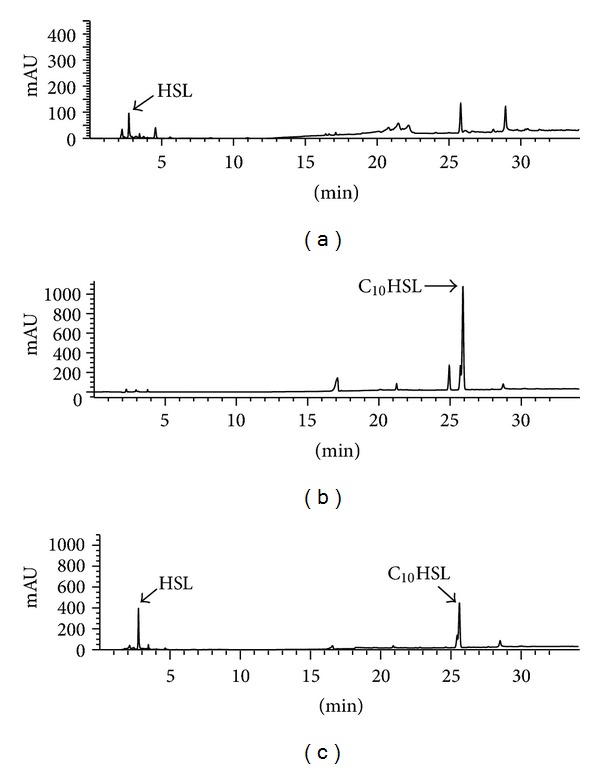
HPLC analysis of enzymatic hydrolysis product of C_10_HSL with the HSL-degrading enzyme from LPC029. The initial C_10_HSL (35 mM) was reacted with partially purified enzyme from LPC029 for 16 h and separated with HPLC. HPLC profiles of (a) HSL standard (3.5 mM); (b) unreacted C_10_HSL (35 mM); and (c) reaction product of C_10_HSL with LPC029 HSL-degrading enzyme. The product peak was eluted at 2.8 min. mAU is the abbreviation for milli-absorbance unit.

**Figure 2:**
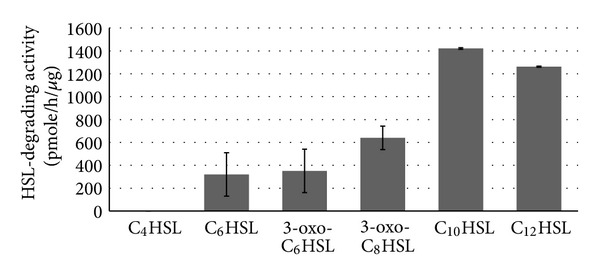
Substrate specificity of LPC029 HSL-degrading enzyme. Partially purified enzyme was mixed with each of HSLs. The activity was determined based on HSL leftover on *C. violaceum* CV026 and *A. tumefaciens* NTL4 (pZLR4) bioassay plates. Relative activity is given in parenthesis. Bars indicate SD values of four replicates.

The products of HSL degradation by enzyme from LPC029 were analyzed by HPLC after ethyl acetate extraction. Hydrolysis of C_10_HSL by LPC029 enzyme resulted in releasing HSL as shown by HPLC (Figure 1(c)) compared to standard HSL (Figure 1(a)) and C_10_HSL substrate alone (Figure 1(b)). Therefore the HSL-degrading enzyme from LPC029 was proven to be HSL-acylase according to the deacylation activity to break the amide bond of C_10_HSL, out of which HSL was released as an end product. The substrate (C_10_HSL) was incompletely hydrolyzed (Figure 1(c)) due to an insufficient amount of enzyme used in the assay. HSL-acylase has been explored in (i) Gram-negative bacteria including *Comamonas* strain D1 [9], *Pseudomonas aeruginosa* PAO1 [30], *P. aeruginosa* PAI-A [31], *P. syringae *[32], *Ralstonia solanacearum* GMI1000 [33], *Shewanella* sp. strain MIB 015 [7], *Tenacibaculum maritimum *[34], and *Variovorax paradoxus* VAI-C [25]; (ii) Gram-positive bacteria including *Rhodococcus erythropolis* strain W2 [35] and *Streptomyces* sp. M664 [8]; and (iii) cyanobacteria such as *Anabaena* sp. PCC 7120 [36]. Comparison of HSL-acylase activity among these HSL-degrading microbes is difficult due to the difference of both quantitative and qualitative measurement methods. Nevertheless, substrate specificity of the enzymes could be compared qualitatively. Although the enzymes were derived from a variety of microorganisms, most of HSL-acylases were active against HSL with the carbon chain less than 8 atoms [10]. Testing of the partial purified enzyme from LPC029 with various HSL substrates showed the ability to degrade medium-to-long chains of HSL (C_6_–C_12_) in which C_10_HSL was the most preferred substrate (Figure 2). The activity of this enzyme was not dependent on the substitution of oxo-group on the third carbon atom (Figure 2) which showed no significant difference between two substrates, C_6_HSL and 3-oxo-C_6_HSL (*P* ≥ 0.05). The results suggested the high possibility of LPC029 HSL-acylase in inhibiting QS of medium-chain HSLs dependent on *Pcc* (3-oxo-C_6_HSL, 3-oxo-C_8_HSL) and *Burkholderia cepacia* (C_6_HSL, C_8_HSL) and might provide the most effective quenching agent against long-chain HSLs produced by *Vibrio anguillarum* (C_10_HSL) and *P. aeruginosa* (C_12_HSL). 

### 3.3. Effect of LPC029 HSL-Degrading Enzyme on Pathogenicity

**Figure 3:**
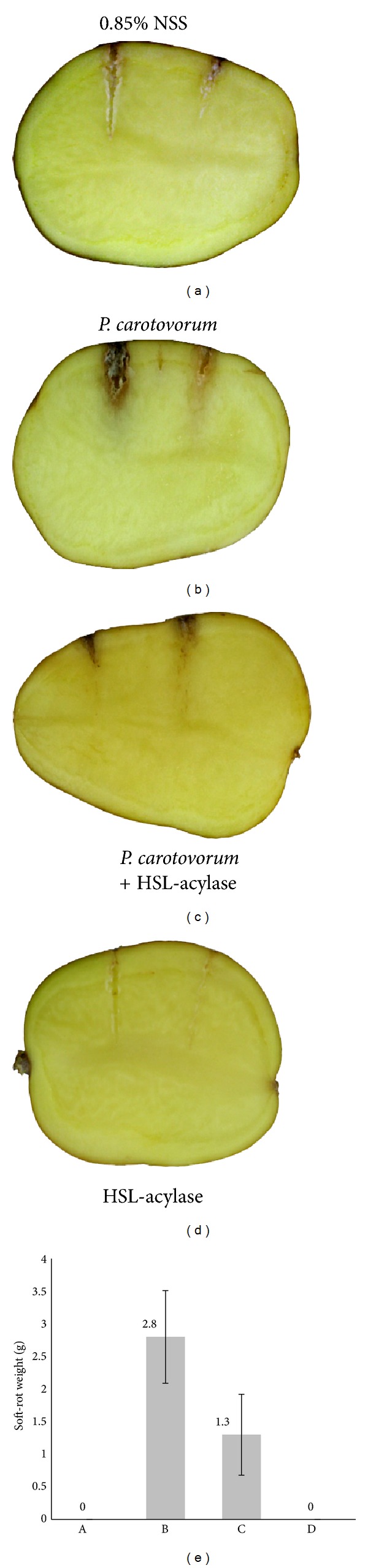
Effects of LPC029 HSL-degrading enzyme on pathogenicity of *Pcc* on potato tubers. Pathogenicity of *Pcc* was determined by inspection of lesion zones induced upon inoculation of potato tuber at two different sites. (a) Negative control consisting of tuber treated with 0.85% NSS, (b) inoculation of *Pcc* alone at 10^3^ cells per tuber, (c) inoculation of *Pcc* at 10^3^ cells mixed with 2 *µ*g of partial purified HSL-acylase per tuber, (d) 2 *µ*g of partially purified HSL-acylase, and (e) pathogenicity of *Pcc* determined by soft-rot weight. Soft-rot tissue from treatment (a)–(d) was measured after inoculation of *Pcc* for 3 days; bars indicate SD values of four replicates.

*In vitro* efficacy of HSL-degrading enzyme from LPC029 to quench QS-regulated functions was determined by *Pectobacterium carotovorum* ssp. *carotovorum* (*Pcc*) mediated soft-rotting potato tuber assay. In *Pcc*, the virulence and pathogenicity are QS dependent, but different strains of this subspecies prefer different signal molecules and they are divided into two groups. One group used 3-oxo-C_6_HSL as a predominant signal molecules, whereas another group exploited mainly 3-oxo-C_8_HSL as HSL-dependent QS [37]. Inoculation of *Pcc* (10^3^ cells per tuber) evidently resulted in tissue maceration. The addition of 2 *µ*g of partially purified HSL-acylase resulted in a statistically significant reduction in soft-rot weight (*P* < 0.05) as shown in Figure 3(e). In the control experiment, neither NSS nor HSL-acylase inoculation induced soft rot in plant tissue (Figures 3(a) and 3(d), resp.). Inoculation of *Pcc* alone (10^3^ cells) caused the highest plant tissue necrosis (Figure 3(b)) with the highest soft-rot weight (Figure 3(e)). When the same number of *Pcc* (10^3^ cells) was mixed with 2 *µ*g of HSL-acylase before inoculation, the enzyme attenuated the bacterial pathogenicity by reduction of plant tissue necrosis (Figure 3(c)) and caused reduction of soft-rot weight (Figure 3(e)). Several studies have shown the effectiveness of QS targeting strategy in reducing virulence of *P. aeruginosa *[38], *Enterobacteriaceae *[39], *B. cepacia *[40], *P. carotovorum *[23, 41, 42], *Serratia liquefaciens *[43], *Agrobacterium tumefaciens *[23], and some aquatic consortia [44]. These results offer an alternative strategy to alternate virulence of plant pathogen using QS-degrading enzyme from endophytic* Streptomyces*. Due to its ability to degrade long-chain HSL with moderate activity toward short-chain HSL, the QQE from *Streptomyces* LPC029 might be of interest to control certain plant and human pathogens. However, more in-depth *in vitro* and also *in vivo* investigations are warranted.

## 4. Conclusion

The HSL-degrading activity among rhizospheric and endophytic actinomycetes was assessed and the latter was found to possess this enzyme activity at higher frequency than the soil isolates. It is interesting to note that this study was the first report on studying the distribution of HSL-degrading enzyme in actinomycetes as well as the first report on finding such an activity in endophytic actinomycetes. Endophytic *Streptomyces *LPC029 showed the highest C_10_HSL-degrading activity at 151.30 ± 3.1 nmole/h/mL and its enzyme showed broad substrate specificity against medium-to-long-chain HSLs. The enzyme was classified as HSL-acylase which hydrolyzes an amide bond between an acyl-side chain and a homoserine lactone and releases a free HSL. The partially purified enzyme from LPC029 attenuated soft-rot diseases caused by *Pcc*, which made the strain as well as many other QQ actinomycetes in this study potential candidates for biocontrol against QS-dependent phytopathogens. Further study on growth conditions including nutrients, temperature, pH, and aerations is required to maximize enzyme productivity in the selected strain(s).

## Figures and Tables

**Table 1:** Relative HSL-degrading activity of actinomycetes isolated from rhizospheric and endophytic samples.

Location	No. of isolates	Relative HSL-degrading activity* (%)						
		0	>0–50	>50–75	>75–87.5	>87.5–93.8	>93.8–96.9	>96.9–100
Rhizospheric samples								
Bangkok	278	182	54	16	11	8	4	3
Chanthaburi	13	6	5	0	0	1	1	0
Nongbualumpoo	3	2	0	0	0	0	0	1
Prachinburi	27	12	9	3	3	0	0	0
Rayong	23	15	8	0	0	0	0	0

Total	344	217	76	19	14	9	5	4

Total (percentage)		217 (63.1%)	127 (36.9%)					

Endophytic samples								
Bangkok	43	29	12	0	1	1	0	0
Nakhonpathom	29	20	8	1	0	0	0	0
Phetchabun	60	15	25	0	10	1	4	5

Total	132	64	45	1	11	2	4	5

Total (percentage)		64 (48.5%)	68 (51.5%)					

*The activity of each isolate was qualitatively determined by comparing with known concentrations of C_10_HSL (200, 100, 50, 25, 12.5, 0.625, and 0 pmole).

**Table 2:** Quantitative HSL-degrading activity of isolates with high efficiency of HSL degradation.

Source	Name of isolate	HSL-degrading activity (nmole/h/mL)
Rhizosphere soil, Nongbualumpoo	PS032	143.42 ± 1.5
Rhizosphere soil, Bangkok	SWP036	144.67 ± 2.1
Rhizosphere soil, Bangkok	SWP042	144.37 ± 1.5
Rhizosphere soil, Bangkok	SWP043	144.26 ± 1.3
Leaf of Ta khram (*Garuga pinnata* Roxb.)	LPC026	146.45 ± 2.8
Leaf of So (*Gmelina arborea* Roxb.)	LPC029	151.30 ± 3.1
Leaf of Kling klang dong (*Stephania venosa *Spreng.)	PC005	146.93 ± 1.0
Leaf of Malabar melastome (*Melastoma malabathricum* L.)	PC052	143.45 ± 4.3
Leaf of Grape-leaf Wood Rose (*Merremia vitifolia* Hallier f.)	PC053	147.68 ± 1.8
